# Strength and Deformation Characteristics of Fiber and Cement-Modified Waste Slurry

**DOI:** 10.3390/polym15163435

**Published:** 2023-08-17

**Authors:** Jiahao Ye, Ping Jiang, Lejie Chen, Xuhui Zhou, Fei Rao, Xinyi Tang

**Affiliations:** 1 School of Civil Engineering, Shaoxing University, Shaoxing 312000, China; 20133328@usx.edu.cn (J.Y.); 21020859007@usx.edu.cn (L.C.); 20020852108@usx.edu.cn (X.Z.); 19133217@usx.edu.cn (F.R.); 19133220@usx.edu.cn (X.T.); 2 Shaoxing Key Laboratory of Interaction between Soft Soil Foundation and Building Structure, Shaoxing 312000, China

**Keywords:** waste slurry, polypropylene fiber, unconfined compressive strength, modulus strength ratio, cumulative strain

## Abstract

Using fiber and cement to modify waste slurry and apply it to roads is an effective way to recycle waste slurry. A new type of road material, fiber–cement-modified waste slurry (FRCS), was prepared in this study. The static and dynamic characteristics of the cement soil were studied using an unconfined compressive strength test and dynamic triaxial test. The results show that the optimum fiber content of FRCS is 0.75%. In the unconfined compressive strength test, under this fiber content, the unconfined compressive strength (UCS) of the FRCS is the largest, and the elastic modulus and modulus strength ratio are both the smallest, indicating that the tensile properties of the cement slurry have been enhanced. In the dynamic triaxial test, the hysteretic curve of the FRCS tends to be stable with the increase in the number of cycles, the dynamic elastic modulus of the FRCS decreases first and then increases with the increase in the dosage, while the damping ratio becomes stable after a rapid decline, and the fiber incorporation increases the cumulative strain of the soil–cement under low-stress cycles, indicating that the ductility of the FRCS is improved. In addition, a cumulative strain prediction model of the FRCS is established in this paper, which can provide a reference for the resource application of waste slurry in road engineering.

## 1. Introduction

With the rapid development of urban construction, a large amount of construction waste such as abandoned slurry is often generated during tunnel shield construction and bored pile construction [1,2]. If not properly disposed of, it will have adverse effects on the environment and natural resources. Using waste slurry as roadbed filler has good prospects and is considered a potential substitute for sand or limestone filler in concrete or mortar [3]. However, without curing measures, the strength of waste slurry after drying is low, and its mechanical properties often do not meet construction requirements. Direct use can lead to defects such as roadbed settlement [4]. There are currently various methods for strengthening and utilizing waste slurry, and the commonly used method is chemical solidification treatment. Cement is added as a chemical stabilizer to the waste slurry to produce a cementitious hardening effect, in order to effectively improve its mechanical properties. Cement, as a commonly used inorganic material, has been widely used both domestically and internationally due to its simple manufacturing process, economic applicability, and low environmental requirements for the site [5,6,7]. Using cement as a curing agent is beneficial for the resource utilization of waste slurry [8,9,10,11]. However, waste slurry mixed with cement alone may have defects such as a low tensile strength, poor crack resistance, and significant cumulative deformation after bearing long-term cyclic loads [12,13]. Moreover, the compounds in the waste slurry lack chemical activity, and the hydration effect is poor after adding cement [14], which limits its promotion and use. Therefore, it is necessary to further reduce the defects of cement slurry to avoid damage to the road structure [15,16].

Adding polymer materials to reduce defects in building materials is an effective way to address them. Fibers, as common polymers, can be used as raw materials for fiber–cement soil, which is a rapidly developing new building material in recent years. Fiber–cement soil has also been a research hotspot for scholars in recent years. Numerous scholars have analyzed the strength and mechanical properties of fiber-reinforced cementitious soil through experiments such as unconfined compressive strength (UCS) testing [17,18,19], and the results show that fibers can effectively improve the strength, durability, and wear resistance of cementitious soil. At the same time, the infinite compressive strength and residual strength of cementitious soil samples with fibers added have also been improved to a certain extent [20]. Polypropylene fibers are commonly used as reinforcing materials for cement-based materials [21], which can improve their mechanical and damage-resistance properties, delay the formation of cracks, and reduce crack width. Jiang et al. conducted a series of experiments to explore the effects of fiber content and fiber length on the strength of fiber-reinforced soil [22]. The research results of Sukontasukkul et al. indicate that as the volume fraction of polypropylene fibers in cementitious soil increases, its toughness also increases [23]. Ruan et al. showed through UCS and flexural strength tests that with an increase in fiber content, the UCS, residual strength, and flexural strength of fiber-reinforced cement mortar soil were significantly improved [24]. Zaimoglu et al. studied the effect of randomly distributed polypropylene fibers (PP) and some additive materials such as borogypsum (BG), fly ash (FA), and cement (C) on the infinite compressive strength of soil. The results showed that PP can effectively improve their infinite compressive strength by combining with additives [25]. Yang et al. explored the reinforcement and stability of loess using fibers as a reinforcement material and cement as a stabilizing material. The results showed that the addition of fibers gradually changed the fracture mode, from brittleness to ductility and then to plasticity [26]. Zhang and others explored the feasibility of the application of polypropylene-fiber-reinforced cement stabilized soil. Through scanning electron microscope (SEM) analysis, the strengthening effect was attributed to the formation of hydration products such as ettringite, the bridging effect, and the increase in particle friction [27]. Chen et al.’s experiment showed that the UCS of fiber–cement admixtures is related to the fiber content and length [28]. Fiber-reinforced cement clay reaches its peak strength at a fiber content of 0.5%. If the fiber content continues to increase, the UCS will slowly decrease. Wang et al. used cement soil mixed with different amounts of polypropylene fibers and basalt fibers to investigate the changes in dynamic stress and dynamic elastic modulus with dynamic strain through dynamic triaxial tests. The results showed that the dynamic characteristics of cement soil were related to the experimental confining pressure, fiber type, and content. With an increase in the fiber content, the dynamic strength and dynamic elastic modulus of cementitious soil increase, while the dynamic deformation decreases [29]. Wang et al. conducted triaxial unconsolidated undrained (UU) tests on polypropylene-fiber-cement-treated roadbed soil (PCS), and the results showed that for the same fiber mass content, the peak stress, residual stress, and peak stress–strain of PCS specimens gradually increased with increasing confining pressure, while the brittleness index gradually decreased [30]. Di et al. conducted dynamic triaxial tests to analyze the effects of high water absorbent polymer (SAP) content, cyclic stress ratio, and loading frequency on SAP–cement-modified soil [31]. Wang et al. conducted triaxial tests to determine the correlation between cumulative plastic strain (CPS) and the number of loading cycles, as well as the evolution of dynamic strength and critical dynamic stress (CDS) with different freeze–thaw cycles, in order to study the dynamic stability of fiber-adhesive-reinforced subgrade fill (RSF) under cyclic loading after freeze–thaw cycles [32].

Meanwhile, the addition of fiber polymers can to some extent improve the problem of road settlement. Excessive settlement and differential settlement of soft foundations are common in roads, and differential settlement can occur at the junction of bridge abutments and roads, as well as issues such as lateral pressure and displacement of bridge abutments [31]. The road is repeatedly subjected to low-stress cyclic loading from traffic loads for a long time, during which the road will not undergo significant damage, but will experience a certain cumulative strain [33]. Factors such as dynamic stress amplitude [34], confining pressure [35], and number of cycles [36] can all affect the generation and development of accumulated strain in the soil, and the bumps caused by deformation can affect the smoothness and safety of vehicle driving [37]. At present, the research on the mechanical properties of FRCS mostly focuses on its static mechanical properties, but its application scenarios are mostly in areas that need to be subject to long-term cyclic low loads, such as roads. Under long-term cyclic loads, reinforced cement soil can limit the lateral displacement of the subgrade and reduce the settlement of the subgrade [38].

Therefore, research on the deformation characteristics of fiber-reinforced cement soil can be of great help for road engineering construction, but there is relatively little research on its cumulative plastic strain characteristics under cyclic loading. This research explores the static and dynamic performance and deformation characteristics of FRCS with different fiber contents and amplitudes through UCS tests and DT tests, in order to provide a theoretical basis for the application of waste slurry in road engineering.

## 2. Materials and Methods

### 2.1. Materials

**Figure 1:**
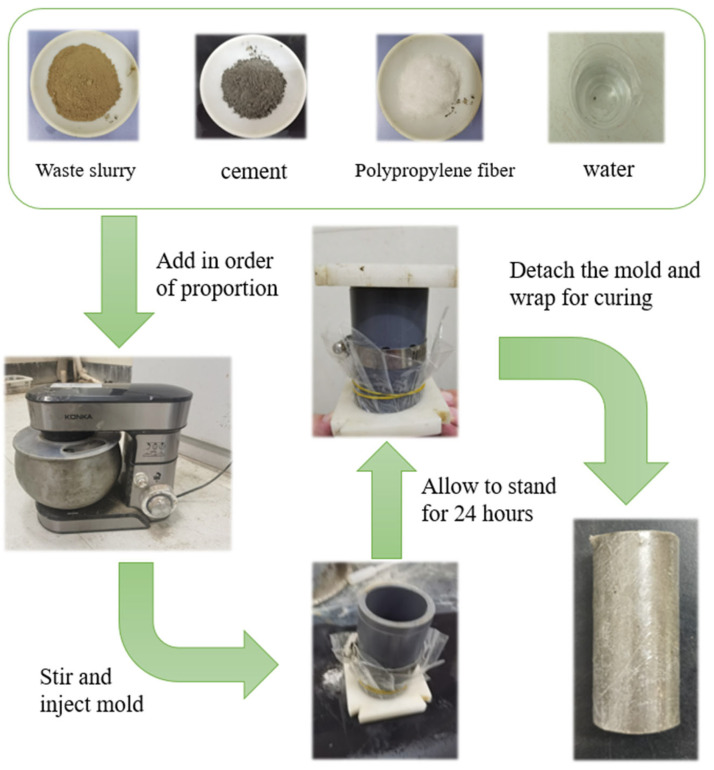
Specimen preparation process.

The materials used in this experiment were waste slurry, cement, and polypropylene fibers. The waste slurry used in the experiment was taken from a construction site in Shaoxing City. The waste slurry after drying is shown in Figure 1; the porosity of the waste slurry was 73%. Its physical and mechanical indicators are shown in Table 1. The main chemical components of the waste slurry, obtained through XRF testing, are shown in Table 2, and the equipment model used was Axiosmax. The main chemical composition of the waste slurry was similar to clay; it did not have chemical activity. The cement used in the experiment was P.O 42.5 ordinary Portland cement, with a density of 3.0–3.15 g/cm^3^ and a specific surface area of 340–370 m^2^/kg. Its porosity was 50%, and its main chemical composition is shown in Table 3. The polypropylene fibers used in the experiment were produced by Shaoxing Fiber High tech Co., Ltd. They have good dispersibility, low cost, and a white color, as shown in Figure 1. Their technical indicators are shown in Table 4.

### 2.2. Specimen Preparation

Each sample was cylindrical, with a diameter of 39.1 mm and a height of a 80 mm. According to the specification for the mix proportion design of cement soil (JGJ/T233-2011) [39], the moisture content was set at 50%, and based on early testing, the ratio of waste slurry, cement, and water was set at a 1:0.1:0.5 mass ratio. The fiber content was proportioned according to the experimental plan.

The specimen preparation process was as follows: (1) Add waste slurry, cement, and water in sequence according to the mix ratio, and add corresponding amounts of polypropylene fibers. (2) Mix thoroughly with a mixer, and after mixing evenly, place them in three layers into the mold and manually vibrate. (3) After vibration compaction and leaving to stand for 24 h, remove the mold and wrap it with cling film to prevent moisture evaporation. (4) Place the specimen in a constant temperature and humidity curing box for curing, with a curing temperature of 20 °C, a humidity of ≥90%, and a curing period of 7 or 28 days. The specific specimen preparation process is shown in Figure 1.

### 2.3. Test Scheme

**Figure 2:**
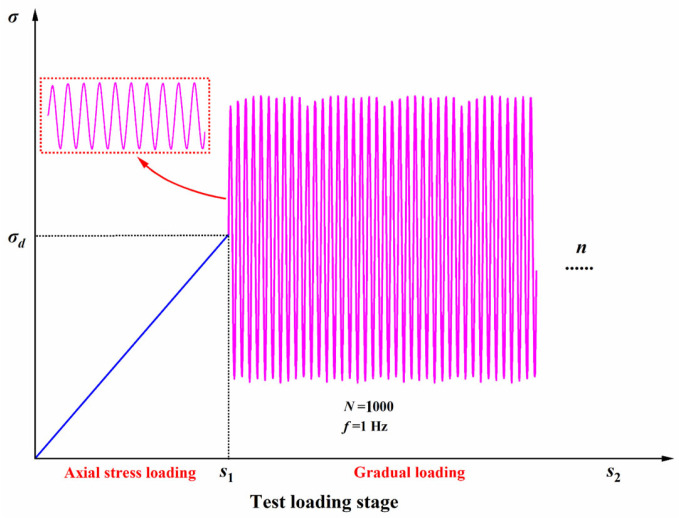
Loading scheme of DT.

The unconfined compressive performance of the FRCS was analyzed using different fiber contents, and the cumulative strain characteristics of the FRCS were explored by applying cyclic loads of different amplitudes. UCS tests were performed on FRCS samples with fiber contents of 0%, 0.25%, 0.5%, 0.75%, and 1% of the dry waste slurry mass. Using strain-controlled loading, according to the Chinese Code Standard for Geotechnical Testing Method (GB/T 50123-2019) [40], the loading rate for the UCS testing was set at 1 mm/min. In the DT test, a sine wave as selected as the loading waveform, and it was loaded using stress as the control method. Twenty points were collected for each cycle, and the specific loading method is shown in Figure 2. By changing the amplitudes of 0.1 UCS, 0.2 UCS, and 0.3 UCS, cyclic loading was performed on the specimens with the optimal fiber content to investigate the effect of the amplitude on the dynamic performance of the FRCS. The specific test plan is shown in Table 5. The UCS test uses the TKA-WCY-1F fully automatic multifunctional unconfined compressive strength tester, and the DT test uses the dynamic triaxial tester produced by GDS Instruments in the UK.

## 3. Results and Discussion

### 3.1. Strength Characteristics

#### 3.1.1. Stress–Strain Curves

**Figure 3:**
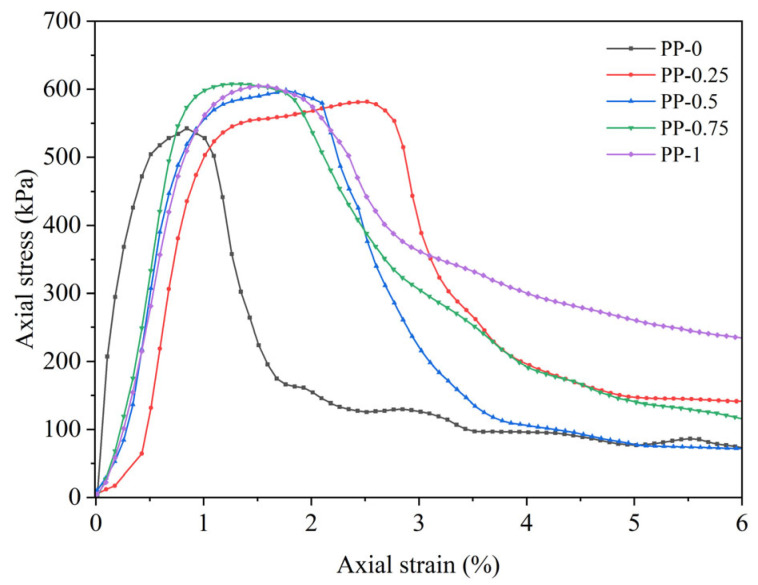
Stress–strain curves of FRCS with 7 d curing age.

**Figure 4:**
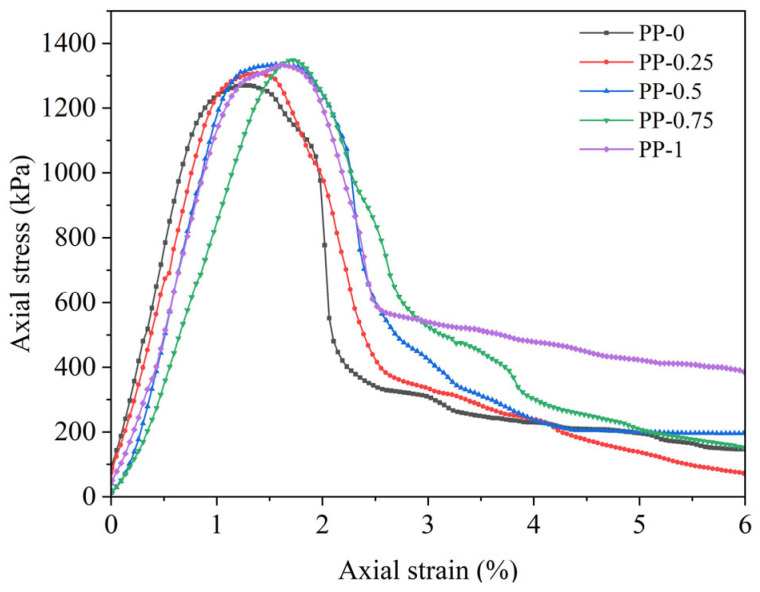
Stress–strain curves of FRCS with 28 d curing age.

UCS tests were conducted on the FRCS with different PP contents, and stress–strain curves were obtained as shown in Figure 3 and Figure 4. The stress–strain curves of the FRCS show almost the same trend under different PP contents, and can be divided into three stages. (1) In the linear elastic stage, the stress increases approximately linearly with strain. (2) As the axial strain continues to increase, it enters the plastic stage, and the stress–strain relationship shows a nonlinear relationship with small fluctuations in the curve. (3) Entering the stress attenuation stage, the stress continuously decreases, and the specimen produces cracks or even failure.

#### 3.1.2. UCS

**Figure 5:**
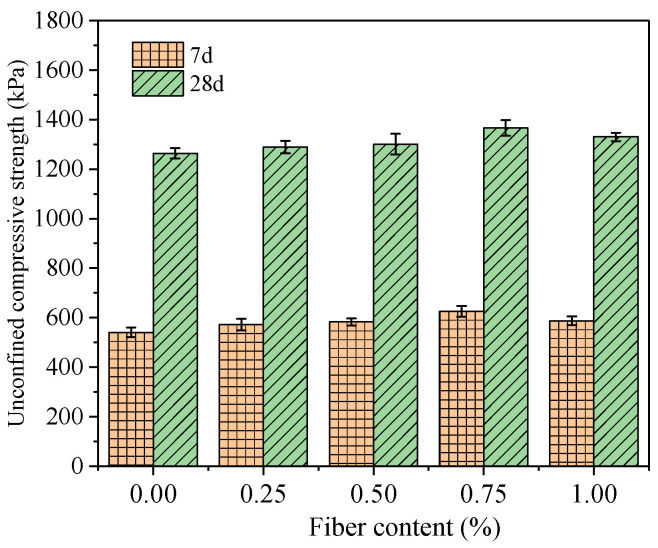
UCS of FRCS at different curing ages.

Figure 5 shows the comparison of the UCS of the FRCS samples with different PP fiber contents. It can be seen from the figure that the UCS of FRCS gradually increases with an increase in the fiber content and reaches its maximum, which is 6.1% higher than that of the specimen without fibers, at a 0.75% fiber content, which is similar to the conclusion in Chen et al. [41]. At the same curing age, the UCS of the FRCS samples with different fiber contents increased in the early stage but decreased to varying degrees in the later stage. This is because as the fiber content increases, its dispersion uniformity in the cement decreases, and the aggregation phenomenon of fibers in the FRCS fails to form an effective spatial network structure. Additionally, certain voids are formed in the FRCS, and the stress transfer ability of the fibers in the soil under load decreases, ultimately leading to a decrease in the UCS of the FRCS. From the overall trend, the UCS of the FRCS containing fibers is higher than that of the FRCS without fibers. PP fibers are helpful in improving the strength of FRCS, and its effect on improving the strength increases with an increase in fiber content. This may be because the waste slurry, cement, and fibers in the specimen produced a bonding force as they harden, allowing the fibers to transmit residual stress and delaying the crack expansion of the FRCS under load, relatively improving the strength of the specimen [42].

From Figure 5, it can also be seen that the UCS of the 28-day curing age specimen is significantly increased compared to the 7-day curing age specimen, being more than double the value. As the curing age increases, the UCS of the specimen increases significantly, which is due to the shorter hydration time of the FRCS cement during the 7-day curing period, resulting in fewer hydration products. However, as the age increases and the hydration reaction continues, a large amount of new minerals, such as calcium salts and aluminum hydrates, in the soil produce crystallization [43,44]. The cement gradually dehydrates over time, resulting in more internal cementation, significantly improving the UCS of the FRCS.

#### 3.1.3. Elastic Modulus

**Figure 6:**
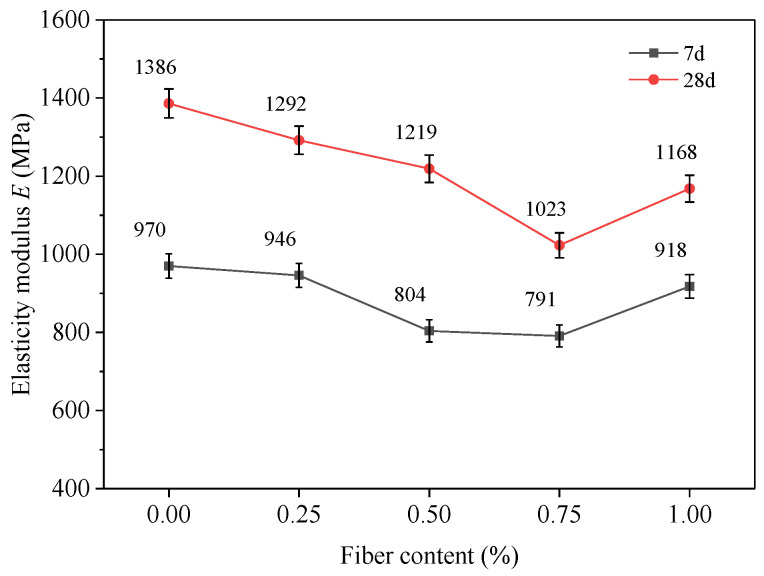
Elastic modulus.

In the UCS test, the stress–strain curves of the FRCS specimens during the elastic deformation stage are positively proportional, and the proportional coefficient is called the elastic modulus *E*, as shown in Figure 6. The smaller the *E* of a material, the greater its elastic deformation, and the easier it is to recover the strain caused by stress. Therefore, materials with a lower elastic modulus are more conducive to improving the variability characteristics of road foundations. From Figure 6, it can be seen that the *E* of FRCS significantly increases from the 7 d to 28 d curing age samples, indicating that its stiffness is significantly increased through sufficient hydration reactions. At both 7 d and 28 d curing age, the samples’ elastic moduli first decrease and then increase with an increase in the fiber content, and the *E* of the sample with 0.75% PP fibers reaches the minimum value.

#### 3.1.4. Ratio of Modulus to Strength

**Figure 7:**
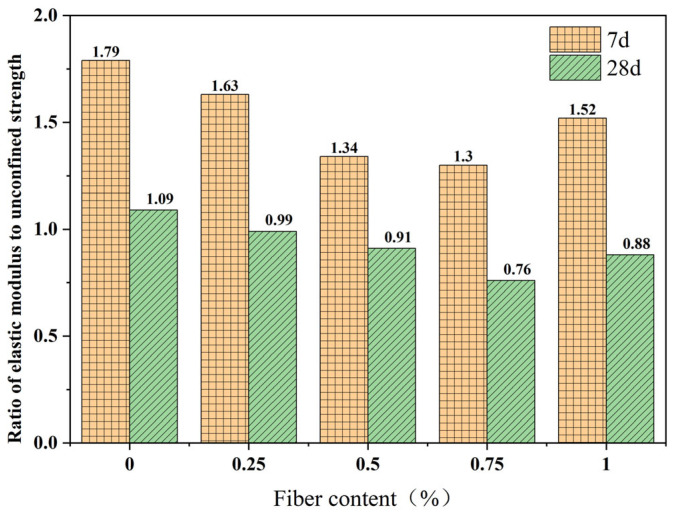
The *η* of FRCS samples.

The ratio of modulus to strength, *η*, is the ratio of *E* to the UCS at peak strength, which can describe the tensile performance of a material. The larger the *η*, the poorer the tensile performance of the material and the lower its ability to resist deformation, as shown in Equation (1). The calculation results are shown in Figure 7.

(1) $$\eta =\frac{E}{UCS}$$

It can be seen from Figure 7 that after adding fibers, the *η* of FRCS decreases, and with the increase in fiber content, the *η* first decreases and then increases. The PP-0.75 specimen reaches the minimum value, and PP-1 then increases. As the curing age increases, the *η* of the FRCS gradually decreases. Taking PP-0.75 as an example, its *η* after 28 days of curing is 58% of that after 7 days, and the *η* has significantly decreased. Therefore, the appropriate fiber content and increasing the curing age can both reduce the *η* of FRCS, thereby improving its tensile performance.

### 3.2. Dynamic Characteristics

**Figure 8:**
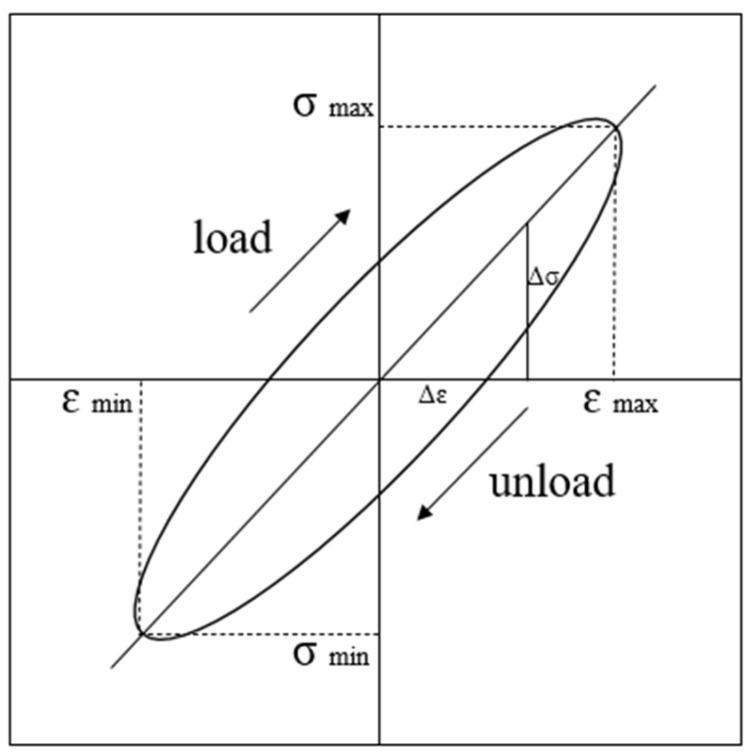
Schematic diagram of hysteresis loop.

In the DT test, the specimen generates a dynamic stress–strain hysteresis loop under the action of cyclic stress during each cycle, and each hysteresis loop curve formed by it generally does not overlap, as shown in Figure 8. The smaller the area of the hysteresis loop of the specimen under the same stress cycle, the smaller the energy loss of the FRCS during this cycle. The dynamic elastic modulus and damping ratio can also be calculated through hysteresis loops.

#### 3.2.1. Hysteretic Curves

**Figure 9:**
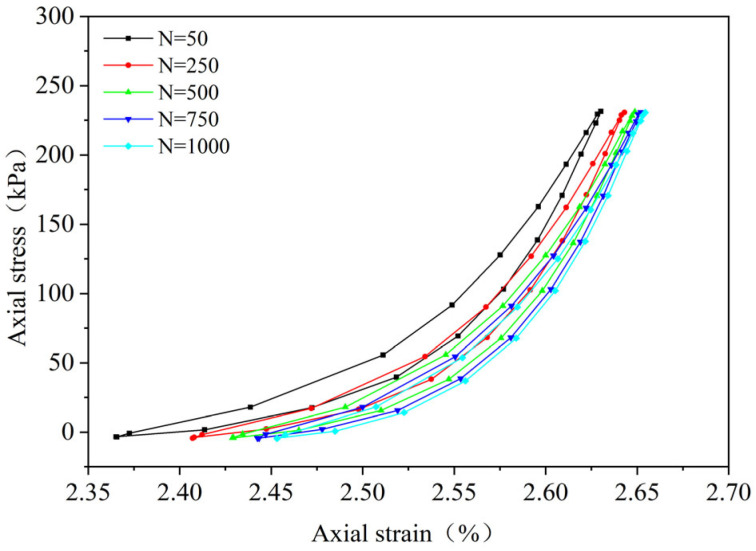
PP-0, 0.1 UCS.

**Figure 10:**
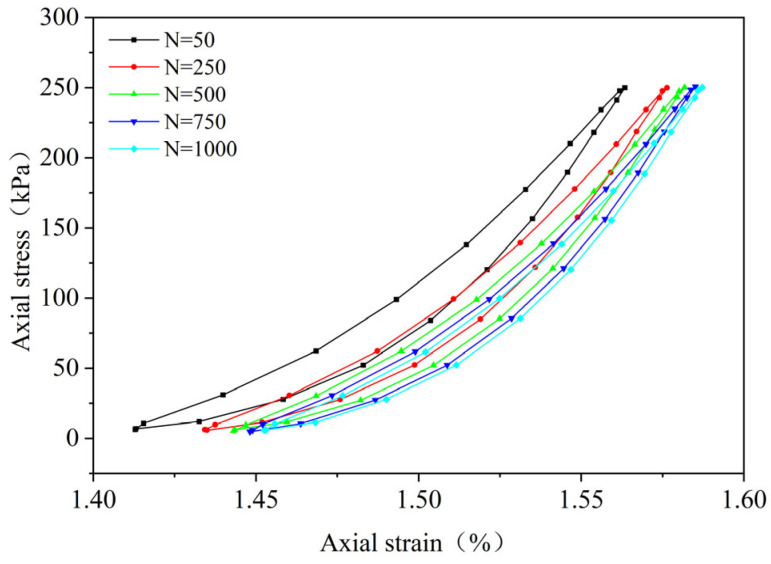
PP-0.25, 0.1 UCS.

**Figure 11:**
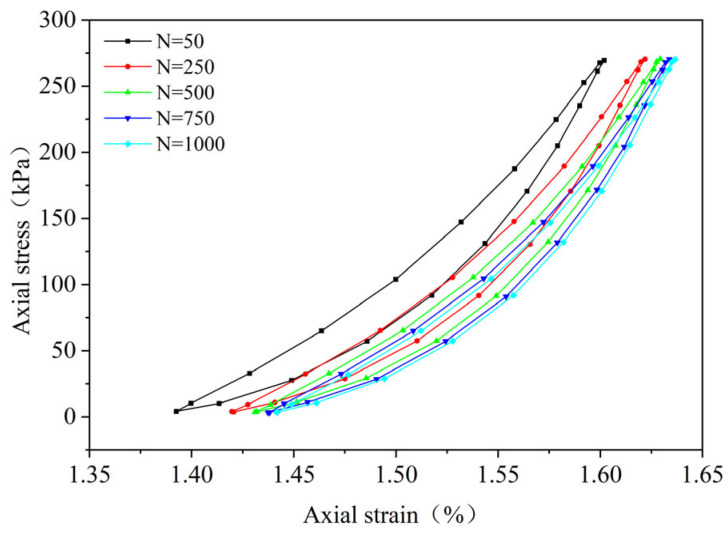
PP-0.5, 0.1 UCS.

**Figure 12:**
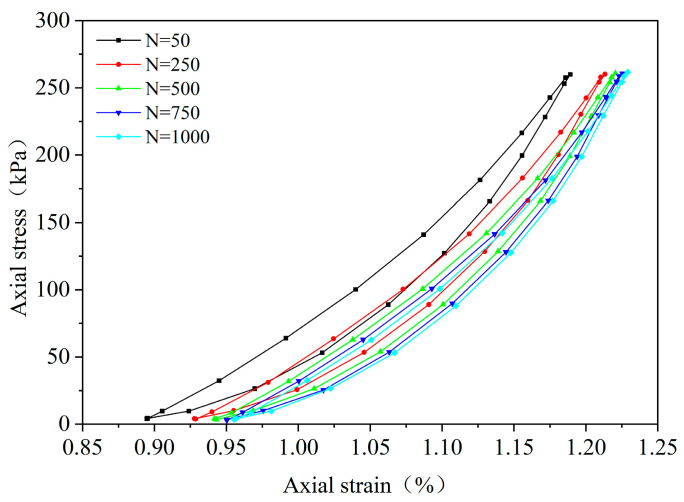
PP-0.75, 0.1 UCS.

**Figure 13:**
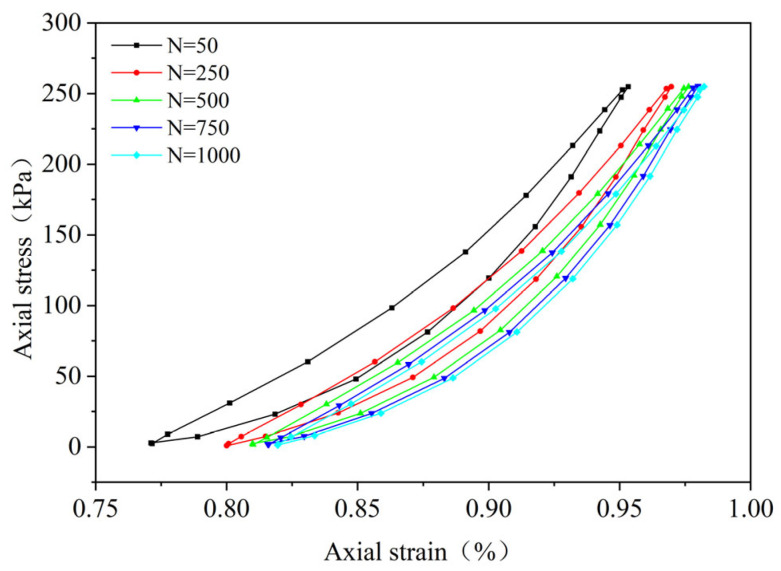
PP-1, 0.1 UCS.

**Figure 14:**
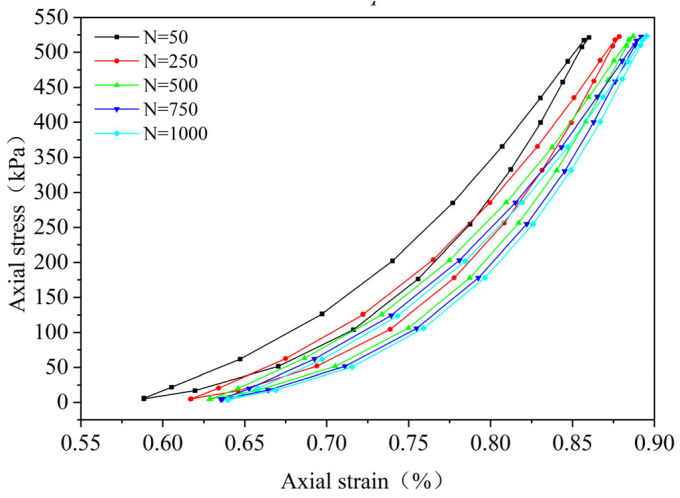
*f* = 0.2 UCS, PP-0.75.

**Figure 15:**
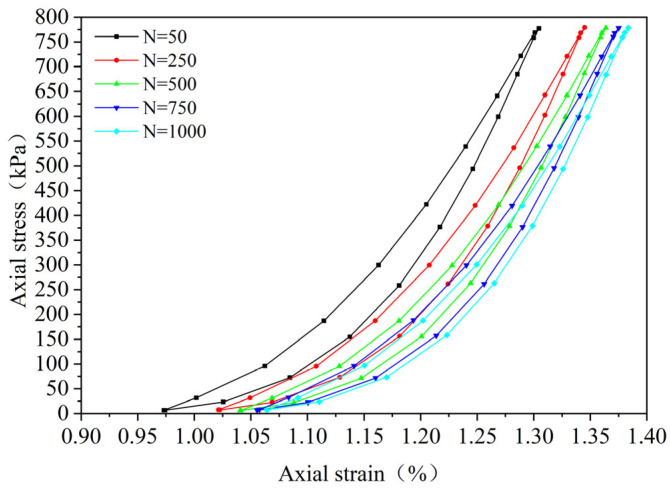
*f* = 0.3 UCS, PP-0.75.

By performing cyclic loading on the FRCS samples with different fiber contents at different amplitudes, hysteresis loops under the 50th, 250th, 500th, 750th, and 1000th loads were selected, as shown in Figure 9, Figure 10, Figure 11, Figure 12, Figure 13, Figure 14 and Figure 15. As the number of cycles increases, the hysteresis loop moves in the direction of increasing strain, indicating that the FRCS generates cumulative strain under the action of cyclic stress. It can be observed that the distance between the hysteresis loops decreases as the number of cycles increases, indicating that the cumulative strain rate gradually decreases to a stable state.

In addition, as the number of cycles increases, the area of the hysteresis loop gradually decreases. The hysteresis loop of unmodified FRCS can be approximated as a closed crescent shape, and the development of hysteresis loops with different fiber dosages and amplitudes has a certain regularity. The development of hysteresis loops with changes in fiber content is shown in Figure 9, Figure 10, Figure 11, Figure 12 and Figure 13. As the fiber content in the soil increases, the length of the hysteresis loops decreases and moves from the direction of the stress axis to the direction of the strain axis. The hysteresis loop of the specimen PP-0.75 reaches its extreme value, while the hysteresis loop of specimen PP-1 is the opposite. The variation in hysteresis loop development with amplitude is shown in Figure 13, Figure 14 and Figure 15. It can be seen that as the amplitude increases, the hysteresis loop becomes narrower and longer. Under the same stress, the larger the amplitude, the smaller the strain. Its shape gradually becomes slender, indicating that the energy loss of the specimen gradually decreases after the start of cycling, and finally tends to a stable state.

#### 3.2.2. Dynamic Elastic Modulus

**Figure 16:**
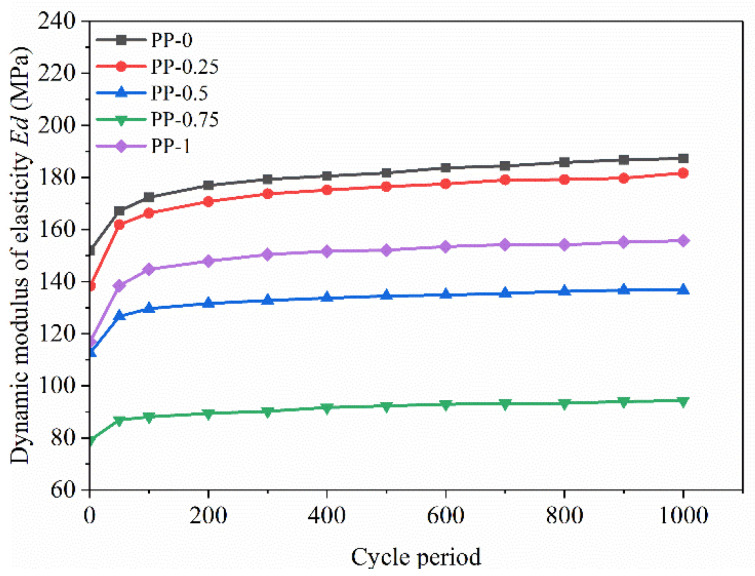
Changes in *Ed* with number of cycles under different fiber contents.

**Figure 17:**
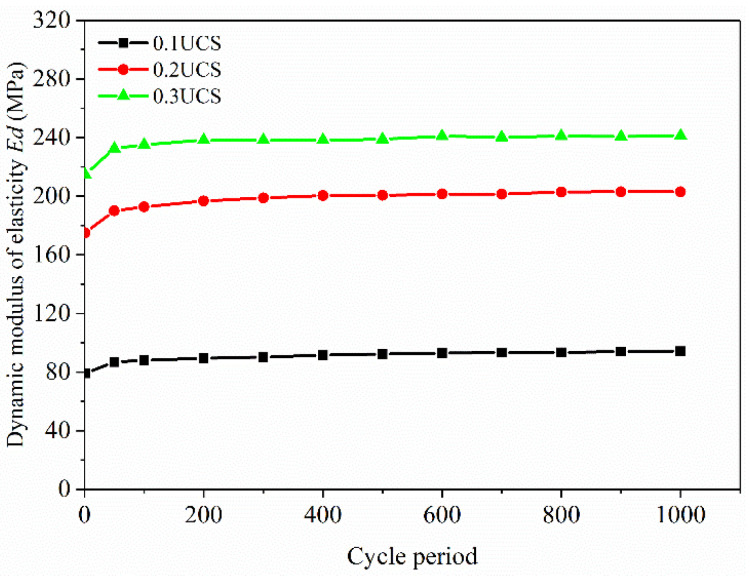
Changes in *Ed* with number of cycles under different amplitudes.

The dynamic elastic modulus *Ed* of the FRCS can be calculated based on its hysteresis loop [20], and the calculation results are shown in Figure 16 and Figure 17.

Figure 16 depicts the relationship between the *Ed* and the number of cycles for each fiber content. It can be seen that as the number of cycles increases, the *Ed* increases rapidly in the first 100 cycles, and then, although it still increases, it tends to flatten out. From the graph, it is found that the *Ed* of the FRCS sample with PP-0.75 changes relatively smoothly, indicating a slower rate of increase in *Ed*. This is because the specimen without fibers is a brittle material which is prone to damage inside the specimen when subjected to external forces. The fibers added will participate in the stress process of the soil and transmit internal stress, which can reduce the internal damage of the specimen. Therefore, the *Ed* of FRCS with fibers added increases slowly. Due to the use of a low cyclic stress ratio, the applied dynamic load is relatively small. When the number of cycles is constant, it is found that with an increase in fiber content, the *Ed* first increases and then decreases, and reaches its maximum for the sample with a PP content of 0.75%. Fibers can have a certain restraining effect on the development of cracks, and the dynamic performance of FRCS can be improved [45].

Figure 17 shows the variation in the *Ed* of the FRCS with different amplitudes during cyclic cycles at a fiber content of 0.75%. It can be seen that the *Ed* of the specimens under different amplitudes significantly increased during the first 100 cycles of cyclic loading. This indicates that during the initial cycles of the FRCS, the Ed of the specimens increases rapidly due to compaction, and later tends to stabilize due to the fact that the specimens have already been compacted, and a more stable structure is formed between the waste slurry, cement, and fibers inside the specimens. The *Ed* tends to stabilize. When the number of cycles is constant, the *Ed* of the FRCS continues to increase and the rate of increase slows down as the amplitude increases. The *Ed* also shows an upward trend with the number of cycles, but the amplitude of the change is small, indicating that under low load cyclic action, the larger the amplitude, the stronger the ability of the FRCS to resist deformation, but the proportion of plastic strain generated will also increase, which is not conducive to the recovery of the internal structure of the soil after compression.

#### 3.2.3. Damping Ratio

**Figure 18:**
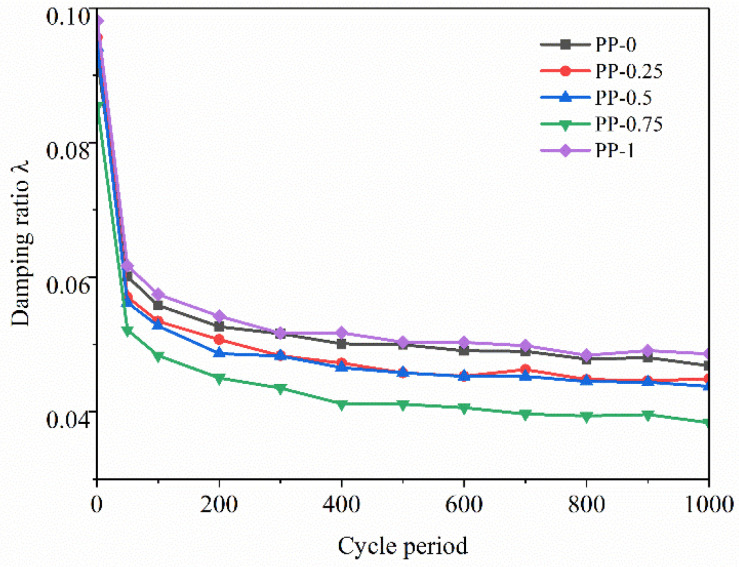
Change in *λ* with number of cycles under different fiber contents.

**Figure 19:**
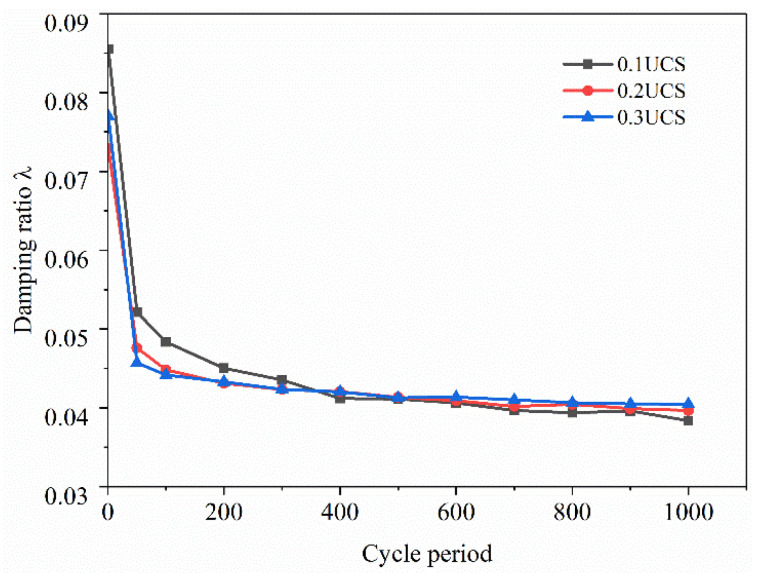
Changes in *λ* with number of cycles under different amplitudes.

The damping ratio *λ* of FRCS can be calculated based on the hysteresis loop [20], and the calculation results are shown in Figure 18 and Figure 19.

Figure 18 depicts the variation in the *λ* of the FRCS samples with different fiber contents over the loading cycles. It can be seen that the amount of fibers added has a certain impact on the *λ* of the specimen, and as the amount of fibers added increases, the impact of dynamic load on the *λ* of FRCS continues to increase. The *λ* reaches its lowest value when the amount of fibers added is 0.75%, indicating that adding an appropriate amount of polypropylene fibers can enhance the compactness of FRCS, improve the overall bearing capacity, and facilitate the internal transmission of stress. Therefore, the energy loss caused during the bearing process is reduced, and *λ* decreases.

In Figure 19, it can be observed that the *λ* of the PP-0.75 specimen increases with the increase in amplitude, leading to more relative sliding and a wider rearrangement of particles inside the FRCS [46]. Under cyclic loading, the internal pores of the specimen are compacted, resulting in a slower energy dissipation rate and a faster rate of reduction in *λ*.

### 3.3. Deformation Characteristics

**Figure 20:**
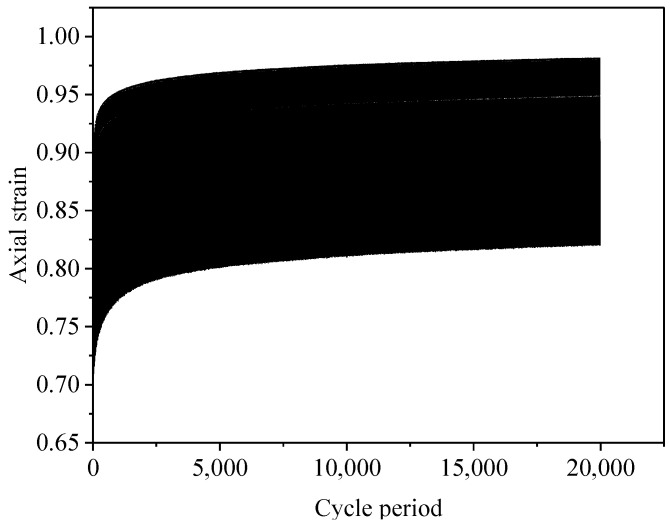
Schematic diagram of the curve of the variation in axial strain with the number of cyclic loading cycles.

Accumulated plastic strain refers to the relative slip of soil particles under cyclic loading, particle rearrangement, and dissipation of the accumulated energy of plastic strain, leading to deformation of the soil. As the number of cycles increases, the dissipation rate of the accumulated energy of viscosity gradually exceeds that of the accumulated energy of plastic strain. At this point, the soil is not damaged and the deformation tends to stabilize. In the daily use of roads, the pore pressure of soft soil in the foundation will first gather and then dissipate under the action of dynamic deviatoric stress under long-term low-stress cycling, during which the soil will generate a certain strain. The cumulative deformation of soil can be divided into three stages: rapid increase stage, gradually stabilising stage, and smooth stage [47]; see Figure 20.

#### 3.3.1. Cumulative Plastic Strain

**Figure 21:**
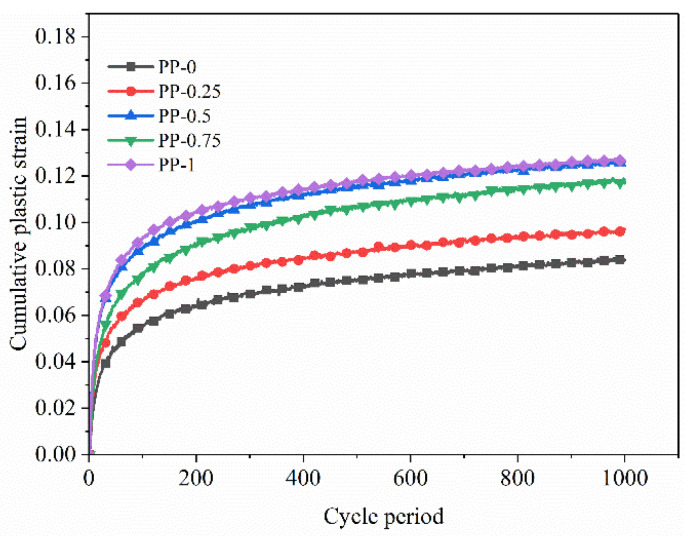
The cumulative plastic strain changes with the number of cycles under different fiber contents.

As shown in Figure 21, the cumulative plastic strain of FRCS with different fiber contents varies with the number of cycles. From the graph, it can be seen that the cumulative plastic strain of FRCS shows a growth pattern of first a rapid increase and then a slow increase with the increase in the number of load applications. With the increase in fiber content, the cumulative plastic strain first increases, then decreases, and then increases. When the fiber content is 0.75%, this is the turning point.

**Figure 22:**
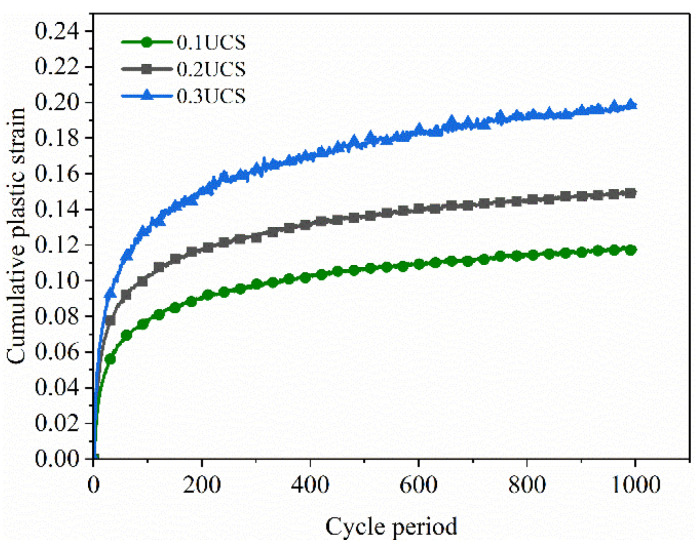
The variation in the cumulative plastic strain with the number of cycles under progressive loading.

Figure 22 shows that the cumulative plastic deformation of FRCS under the same fiber content increases with the increase in amplitude, and the rate of growth also increases with the increase in amplitude. For foundations with the appropriate fiber content in the FRCS, the settlement of the roadbed can be alleviated.

#### 3.3.2. Cumulative Deformation Prediction

From the previous test analysis, it can be found that the cumulative plastic strain of FRCS increases with an increase in the number of cyclic loading cycles, and the logarithm of the cumulative plastic strain and the number of cycles of cyclic loading satisfies a power function relationship, as shown in Equation (2).

(2) $$\varepsilon_{d}=a{\left(\ln N\right)}^{b}$$

where *ε*_d_ is the cumulative plastic strain in %, *N* is the number of cycles of loading, and *a*, *b* is a parameter related to the stress amplitude and fiber content.

**Figure 23:**
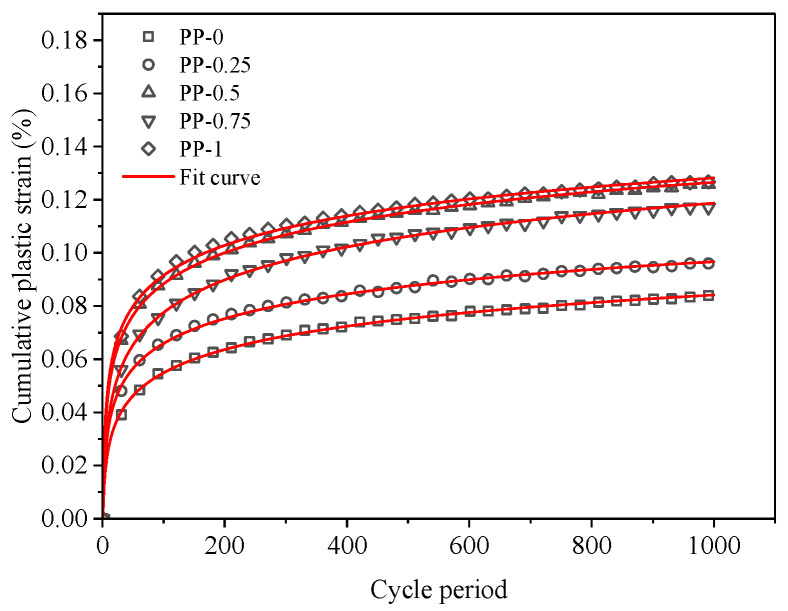
Cumulative plastic strain fitting results of FRCS under different fiber dosages.

**Figure 24:**
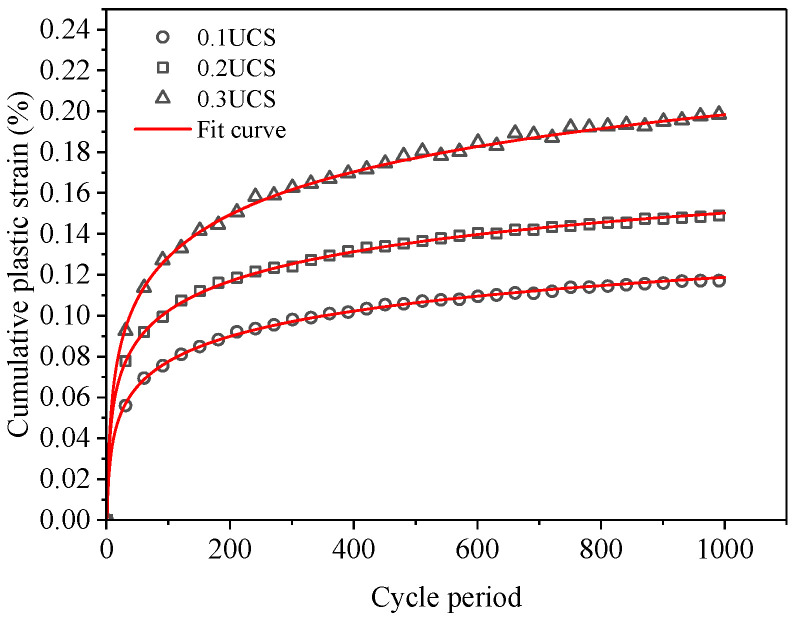
Cumulative plastic strain fitting results of FRCS under different stress amplitudes.

By using Equation (2) to fit and calculate the cumulative plastic strain curve of FRCS, the relevant parameters shown in Table 6 can be obtained, and the fitting curves are shown in Figure 23 and Figure 24. It was found that the theoretical and experimental values have a small error, and this function can better describe the relationship between the cumulative plastic strain of FRCS and the number of cyclic loading cycles.

## 4. Conclusions

The strength and deformation characteristics of FRCS were studied through unconfined compressive strength tests and dynamic triaxial tests, and the following conclusions were drawn:

(1) The UCS and static elastic modulus of FRCS can be improved with an increase in the fiber content, and the optimal fiber content is 0.75%. With the increase in fiber content, both the UCS and static elastic modulus of the sample decrease first and then increase. Moreover, the appropriate amount of fibers can effectively reduce the modulus strength ratio of FRCS and improve its tensile properties.

(2) The hysteresis curve of FRCS tends to be stable with an increase in the number of cycles, and its shape and size are affected by the fiber content and the amplitude. The addition of fibers can reduce the Ed of the sample, and the Ed increases rapidly with the increase in the number of cycles and then tends flatten out. The Ed of FRCS first decreased and then increased with the increase in the fiber content, and the Ed of FRCS continued to increase with an increase in amplitude. With the increase in the number of cycles, the damping ratio of FRCS showed a linear rapid decrease, and then slowed down and stabilized in a small range. The Ed and the damping ratio of FRCS are optimal when the fiber content is 0.75%.

(3) With an increase in the fiber content, the cumulative plastic strain of FRCS increases first and then decreases under the same number of cycles, and the cumulative plastic strain reaches a maximum value when the fiber content is 0.75%. The cumulative plastic deformation and growth rate of FRCS with the same fiber content increase with an increase in amplitude. The cumulative strain of FRCS increases rapidly during the initial cycles, but becomes stable after more than 250 cycles. A cumulative plastic strain prediction model of FRCS is established, and the logarithm of cumulative plastic strain and cyclic loading times satisfy the power function relationship.

## Figures and Tables

**Table 1:** Basic mechanical performance indicators of waste slurry.

Index	Specific Gravity	Liquid Limit/%	Plastic Limit/%	Plasticity Index/%	Moisture Content/%
Value	2.68	42.4	21.9	20.5	100

**Table 2:** Chemical composition of waste slurry.

Composition	SiO_2_	Al_2_O_3_	Fe_2_O_3_	CaO	K_2_O	MgO	Others
Content/%	63.28	18.88	5.89	3.96	2.82	2.45	2.73

**Table 3:** Chemical composition of cement.

Composition	CaO	SiO_2_	Al_2_O_3_	Fe_2_O_3_	SO_3_	MgO	Others
Content/%	47.6	26.8	7.5	5.9	4.1	2.9	5.2

**Table 4:** Main performance indicators of polypropylene fibers.

Specific Gravity	Diameter/µm	Tensile Strength/MPa	Elastic Modulus/GPa	Tensile Limitv /%
0.91	48	385	4.2	15–18

**Table 5:** Test scheme.

Test	NO	Fiber Content (%)	Amplitude (kPa)	Loading Rate (mm/min)	Frequency (Hz)	Number of Vibrations
UCS	PP-0	0%	—	1 mm/min	—	—
	PP-0.25	0.25%	—			
	PP-0.50	0.5%	—			
	PP-0.75	0.75%	—			
	PP-1	1%	—			
DT	PP-0	0%	0.1 UCS	—	1	1000
	PP-0.25	0.25%	0.1 UCS			
	PP-0.50	0.5%	0.1 UCS			
	PP-0.75	0.75%	0.1 UCS 0.2 UCS 0.3 UCS			
	PP-1	1%	0.1 UCS			

**Table 6:** Cumulative plastic strain prediction parameter values.

PP Fiber Content/%	Stress Amplitude/kPa	*a*	*b*	*R* ^2^
0	0.1 UCS	0.01097	1.05438	0.99
0.25		0.01556	0.94541	0.99
0.5		0.0228	0.88687	0.99
0.75		0.01556	1.05155	0.99
1		0.02569	0.83172	0.99
0.75	0.1 UCS	0.01556	1.05154	0.99
	0.2 UCS	0.02408	0.94723	0.99
	0.3 UCS	0.02517	1.06808	0.99

## Data Availability

Not applicable.
